# Population genetic structure of *Anopheles arabiensis *and *Anopheles gambiae *in a malaria endemic region of southern Tanzania

**DOI:** 10.1186/1475-2875-10-289

**Published:** 2011-10-05

**Authors:** Kija R Ng'habi, Bart GJ Knols, Yoosook Lee, Heather M Ferguson, Gregory C Lanzaro

**Affiliations:** 1 Biomedical and Environmental Thematic Group, Ifakara Health Institute, Box 53, Ifakara, Tanzania; 2 Division of Infectious Diseases, Tropical Medicine & AIDS, Academic Medical Center, F4-217, Meibergdreef 9, 1105 AZ Amsterdam, The Netherlands; 3 K&S Consulting, Kalkestraat 20 6669 CP Dodewaard, The Netherlands; 4 Department of Pathology, Microbiology and Immunology, School of Veterinary Medicine, UC Davis, USA; 5 Institute of Biodiversity, Animal Health and Comparative Medicine, University of Glasgow, G12 8QQ, Glasgow, UK

## Abstract

**Background:**

Genetic diversity is a key factor that enables adaptation and persistence of natural populations towards environmental conditions. It is influenced by the interaction of a natural population's dynamics and the environment it inhabits. *Anopheles gambiae s.s. *and *Anopheles arabiensis *are the two major and widespread malaria vectors in sub-Saharan Africa. Several studies have examined the ecology and population dynamics of these vectors. Ecological conditions along the Kilombero valley in Tanzania influence the distribution and population density of these two vector species. It remains unclear whether the ecological diversity within the Kilombero valley has affected the population structure of *An. gambiae s.l. *populations. The goal of this study was to characterise the genetic structure of sympatric *An. gambiae s.s *and *An. arabiensis *populations along the Kilombero valley.

**Methodology:**

Mosquitoes were collected from seven locations in Tanzania: six from the Kilombero valley and one outside the valley (~700 km away) as an out-group. To archive a genome-wide coverage, 13 microsatellite markers from chromosomes X, 2 and 3 were used.

**Results:**

High levels of genetic differentiation among *An. arabiensis *populations was observed, as opposed to *An. gambiae s.s.*, which was genetically undifferentiated across the 6,650 km^2 ^of the Kilombero valley landscape. It appears that genetic differentiation is not attributed to physical barriers or distance, but possibly by ecological diversification within the Kilombero valley. Genetic divergence among *An. arabiensis *populations (*F*_ST _= 0.066) was higher than that of the well-known M and S forms of *An. gambiae s. s. *in West and Central Africa (*F*_ST _= 0.035), suggesting that these populations are maintained by some level of reproductive isolation.

**Conclusion:**

It was hypothesized that ecological diversification across the valley may be a driving force for observed *An. arabiensis *genetic divergence. The impact of the observed *An. arabiensis *substructure to the prospects for new vector control approaches is discussed.

## Background

The genetic structure of a population is shaped by interactions between the behaviour of individuals and their prevailing environment [1]. These factors in combination, can influence the magnitude of gene flow within and between populations and the genetic structure of populations of a species throughout its range [2]. Ecological diversification has been suggested to be one of the factors that can interrupt the movement of genes by creating landscape or genetic barriers such as rapid chromosomal evolution that may ultimately result in reproductive divergence and speciation [3]. Understanding the relationship between individuals and their surrounding environment can, therefore, provide information about the movement of genes from one individual/population to another. This information is important not only for understanding species evolution, but also, in the case of disease vectors, for their control.

The method commonly used to study patterns of gene flow includes identifying genetic units within an ecological landscape and the features that help to shape the spatial distribution of these units [4]. This involves describing genetic boundaries among pre-selected collection sites, presumed to be Mendelian populations, and estimating levels of gene flow among them using *F*_ST _values or other parameters. Using this information it may be possible to deduce which features/factors are responsible for restricting or promoting movements of genes between or within these populations [5,6]. Although this method has been useful, it suffers from drawbacks. It may for example, not be informative for small areas and relies on prior assumptions of population limits [4,7,8]. The development of Bayesian clustering methods have proven useful since they use individual genotypes as a sole source of information and individuals can be partitioned into genetic units with genotype frequencies in Hardy-Weinberg equilibrium [9]. Bayesian clustering methods have gained popularity and have been applied to population genetic studies of a range of organisms such as humans [10], animals [4,11] and plants [10].

Mosquitoes of the *Anopheles gambiae *complex, include the two primary vectors (*An. gambiae s.s. *and *Anopheles arabiensis*) of human malaria in sub-Saharan Africa, that are responsible for an estimated 240 million cases and 280,000 deaths worldwide, with over 80% occurring in Africa [12]. These two species are the most widespread members of the *An. gambiae *complex and major vectors of malaria [13]. Although they are commonly found occupying similar ecological niches, *An. gambiae s. s. *is associated with more humid climates than *An. arabiensis*, which has a greater tolerance for drier environments [14,15]. Additionally, *An. gambiae s.s. *are highly anthropophagic [13,16], endophagic and typically endophilic [16], whereas *An. arabiensis *are more zoophagic, exophagic [17], and exophilic [18,19]. A strong pre-copulatory barrier exists between the two species. Although the post-mating isolation mechanism is incomplete, hybrids which are fertile [20] are competitively inferior as evidenced by rare hybrids in nature (0.02-0.76%) [6,21].

Population substructure is more pronounced in *An. gambiae s. s *and is thought to be influenced by environmental heterogeneity [22]. For example based on chromosomal inversions, five distinct *An gambiae s.s. *subpopulations, which exist in sympatry, have been revealed in West and Central Africa [6,23]. Studies from north, south and western Africa have reported some degrees of genetic differentiation between *An. arabiensis *populations [24-26]. However, neither physical barriers nor geographic distance has been reported to be forces responsible for *An. arabiensis *population differentiation [26,27], except for island populations whose genetic differentiation has been associated with historical drifts [24].

*Anopheles gambiae s.s. *subpopulations have been studied intensively and are of great epidemiological importance as they have been suggested to undermine available malaria vector control efforts. For example the two molecular forms (S and M) of *An. gambiae s.s*. have been reported to respond differently to control interventions, as the S form has developed resistance to pyrethroids (insecticides used for impregnating bed-nets) while the M form remains largely susceptible [28,29]. In addition to undermining current control interventions, such population subdivisions are expected to pose more challenges to the application of new genetic control approaches. Such population subdivision may require genetic modification of multiple strains for successful introduction and spread of desired traits into wild populations [5,18]. Therefore, understanding the genetic structure and relative amount of gene flow taking place within and among wild populations is an important component for effective planning and implementation of available insecticide-based vector control approaches. Additionally, poor understanding of the genetic structure and level of gene flow between target populations may possibly undermine proposed genetic control strategies, especially those that aim at reducing mating success of genetically-modified and/or sterile male mosquitoes from natural populations [30,31]. The existence of genetic substructure within vector populations would create barriers that may restrict the spread of desired genes [5,32].

This study, characterised the population structure of *An. gambiae s.s*. and *An. arabiensis *within the Kilombero valley (6650 km^2^) located in southern Tanzania. Although there are remarkable reductions in transmission intensities, this valley experienced some of the most intense malaria transmission in the world [33,34]. Epidemiological studies in the valley revealed that, malaria transmission intensities, as indexed by entomological inoculation rate (EIR, number of infective bites a person is exposed to in a year) are very high and range between 100 to 1000s of infective bites per annum [33,35-37]. It remains unclear whether such high levels of transmission can be attributed to environmental factors that affect mosquito population density and distribution within the valley [38,39] or whether genetic factors that increase vectorial capacity play a larger role. Several studies have examined the ecology and population dynamics of malaria vectors within the Kilombero Valley [36,38,39], but there are no studies of the population structure of *An. gambiae s. s. *and *An. arabiensis *in it. In this study therefore, a Bayesian clustering analysis was used and 13 polymorphic microsatellite loci originally designed for use in *An. gambiae s. s. *[40] were employed to characterise the genetic structure of each of the two malaria vectors throughout the Kilombero Valley. The hypothesis that physical distance between collection sites may affect gene flow was tested and discrete genetic units within each of the two species were created.

## Methods

### Study site and mosquito collection

**Figure 1:**
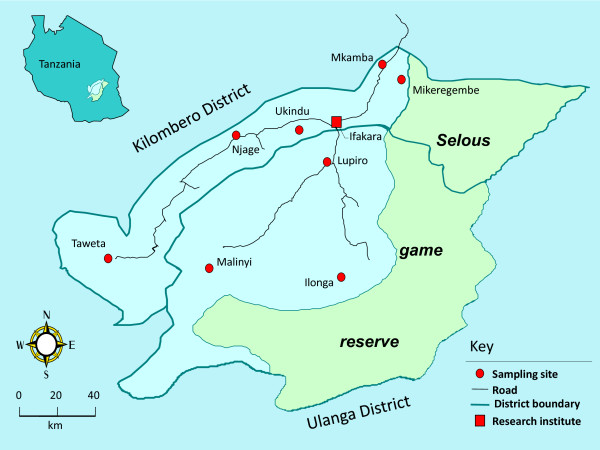
**Map of the Kilombero valley**. The map of the Kilombero valley (Tanzania), showing mosquito collection sites.

The Kilombero Valley is located in south-eastern Tanzania (Figure 1). The two major vectors of malaria, *An. gambiae s.s. *and *An. arabiensis*, are widely distributed along the valley, which is oriented from the northeast to southwest (7°44'-9°26'S/35°33'-36°56'E), The valley lies between the densely forested escarpment of the Eastern Arc mountains and covers an area of 6650 km^2 ^[41]. Estimated mean annual rainfall ranges from 1200 mm/yr to >2000 mm/yr [39,42]. Mosquitoes were collected from seven localities from January - May 2007. Two locations are situated in close proximity with the forested Eastern Arc mountains (Mkamba and Ilonga) and four locations were situated along the river (Malinyi, Ukindu, Lupiro, and Mikeregembe). The distance between these collection sites ranged from 20 to 50 km. Mosquitoes were also collected from Kaliua village located **>**700 km west of the Kilombero Valley, as an out-group. In each village, five CDC light traps were placed in different randomly selected houses for three consecutive nights. Every morning, traps were retrieved and mosquitoes identified according to their morphological differences. Mosquitoes visually identified as belonging to the *An. gambiae *complex were individually stored on silica gel in Eppendorf tubes and taken for further molecular analysis to determine whether they were *An. gambiae s.s. *or *An. arabiensis*.

### DNA extraction and species identification

Prior to DNA extraction, individual mosquitoes were rehydrated in 100 μl of ultra pure water overnight. The following day, 10 μl of proteinase K and 90 μl of ATL buffer were added to each individual tube, and homogenized in a Qiagen Tissue Lyser^®^. DNA extraction was performed using a Qiagen 96 Biosprint^® ^work station in 96 well plates using a BioSprint96 DNA Blood Kit (Qiagen, CA). Each individual of the *An. gambiae *complex was identified to species level using species-specific PCR primers [43].

### Microsatellite DNA amplification

We screened PCR primers for 20 *An. gambiae *microsatellite loci [40]. These were initially developed for *An. gambiae s.s*., and only 13 gave good amplification in both *An. gambiae s.s. *and *An. arabiensis*. Consequently only these 13 were used in this study. These markers provide representative coverage of the entire genome as they are distributed relatively evenly throughout the genome as follows: X chromosome: *AGXH25*, *AGXH100 *and *AGXH71*; Chromosome 2: *AG2H175*, *AG2H85*, *AG2H164*, *AG2H197 *and *AG2H675*; and Chromosome 3: *AG3H127*, *AG3H249*, *AG3H311*, *AG3H811 *and *AG3H9 *[40].

Microsatellite loci were PCR-amplified for each mosquito sample. Each 25 μl PCR reaction consisted of 12.5 μl of Multiplex master mix, 2.5 μl primer mix and 9 μl of RNase free water. The primer mix was made to a final volume of 250 μl, consisting of 10 pmol of each primer. The forward primer in each reaction was labeled with a fluorescent marker (FAM, NED or HEX) compatible with ABI PRISM (Perkin-Elmer, Norwalk, Conn.) capillary electrophoresis. DNA amplification was completed in MJ Research PTC-200 thermal cyclers (MJ Research, Watertown, MA). A 5 min denaturation step at 95°C was followed by 29 cycles of 20 s at 95°C, 30 s at 55°C and 30 s at 72°C. A final incubation at 72°C was extended for 1 hour to alleviate problems associated with addition of non-template nucleotide (dA) to the PCR products. PCR products were mixed with a GeneScan (Perkin-Elmer, Norwalk, CT) size standard and deionized formamide as directed by the manufacturer. Mixtures were run on an ABI 3130 Genetic analyzer. Output was analysed using ABI PRISM^® ^3130 Genemapper (Applied Biosystems) to identify alleles.

### Statistical analysis of microsatellite allele frequencies

Microsatellite allele and genotype frequencies were examined using *Arlequin *[44] developed by Excoffier and colleagues [45]. Each microsatellite locus was tested separately for significant departure from Hardy-Weinberg equilibrium, using a Markov-chain algorithm [46]. Significance threshold were adjusted for multiple comparisons using the formula 1-(1-0.05) ^ (1/n), where n is the number of independent comparisons [47]. The *Arlequin *software was used to calculate pair-wise *F*_ST _values [45], and 10,000 permutations were used to determine the significance of *F*_ST _distance. The neighbour-joining algorithm implemented in *neighbour*, a part of the *Phylip *software package [48] was used to calculate the unrooted tree based on the matrix of pair-wise *F*_ST _distances. The cladogram was drawn using the *drawtree *programme, also provided in *Phylip*. As an estimate of gene flow, the number of migrants per population per generation (*Nm*) was calculated for *F*_ST _according to the equation, Nm ≈ (1-*F*_ST_)/4*F*_ST _[49]. A Bayesian clustering analysis was applied based on the 13 microsatellite markers on chromosomes X, 2 and 3. Using *structure *software [9], individuals that share the same alleles (unique to that group) are placed into groups/clusters termed as (*K*), chosen in advance. This model calculates the probabilities of each individual for each subgroup, within which Hardy-Weinberg (H-W) equilibrium and linkage equilibrium are met. These probabilities are used to infer the membership of each individual at their most probable subgroup, and these are referred to as *membership coefficients *which sum to 1. The probability distribution of an individual X in a putative population K (P(X/K)) was also plotted. Based on allele diversity, individuals with unique alleles are grouped together into assumed population (*K*) which is pre-determined. The *K *value with the maximum posterior probability (P(X/K)) is retained and assumed to be the most probable number of clusters in that putative population.

## Results

A total of 603 female *An. gambiae s. l. *mosquitoes were collected in this study from the seven localities. Of these, 113 (18.7%) were *An. gambiae s. s. *and 490 (81.2%) were *An. arabiensis*. A total of 386 (288 *An. arabiensis *and 98 *An. gambiae s. s.*) mosquitoes were screened for the 13 microsatellite loci.

### Genotype frequencies

The result of Guo's Exact Hardy-Weinberg test [46] are shown in Table 1. There was substantial departure from H-W expectations: 22 out of 78 tests in *An. arabiensis *(28%) and 10 out of 39 tests in *An. gambiae s.s. *(25.6%) were significant (*P *< 0.00044). Further inspection in *An. arabiensis *populations revealed that nine out of 21 tests (42%) involving X-linked loci significantly deviated from H-W expectations and no test out of nine tests (0%) in *An. gambiae s.s. *significantly deviated from H-W expectations (Additional file 1) suggesting subdivision or admixture within *An. arabiensis *populations in the Kilombero Valley.

### Linkage disequilibrium

**Figure 2:**
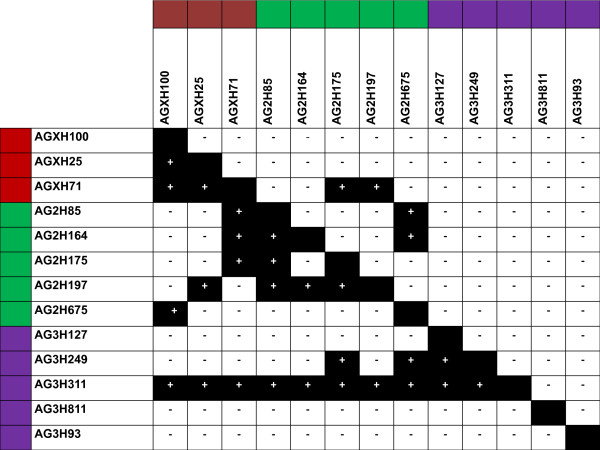
**Linkage disequilibrium**. A pairwise linkage disequilibrium for 13 microsatellite loci for *An. gambiae s.s*. (above diagonal) and *An. arabiensis *(below diagonal). Black boxes with "+" indicates statistical significant at linkage disequilibrium (*P *< 0.00033). The " - " indicates a no significant linkage disequilibrium (*P *< 0.00033).

The non-random association between polymorphism at different loci is measured by the degree of linkage disequilibrium. Linkage disequilibrium was determined [49] and 33.3% of the overall pairwise comparisons among *An. arabiensis *populations were statistically significant (*P *< 0.00066), (Figure 2). In *An. gambiae s.s*. however, there was 5% overall pairwise comparison observed to be significant (*P *< 0.00066). It has been established that linkage disequilibrium is predicted to approach zero for an ideal population, in the absence of forces such as genetic drift, population mixing, mutation and natural selection [50]. It was, therefore, hypothesized that the high linkage disequilibrium 33% observed for *An. arabiensis *suggests the existence of population subdivision [51].

### Population structure

Differences in allele frequencies among populations were described using *F*_ST _estimates as a measure of differentiation between the two species and among populations. Results are presented in Table 2. *Anopheles arabiensis *was genetically distant from *An. gambiae s.s. *(*F*_ST _ranging from 0.21-0.27) (Additional file 2). The level of genetic divergence between *An. gambiae *populations was low (*F*_ST _ranging from 0.003 to 0.01) whereas the level of genetic divergence between *An. arabiensis *populations was substantially higher (*F*_ST _ranging from 0.006 to 0.1). The product of effective population size *N*_*e *_and the migration rates *m *was used to estimate the amount of gene flow within each species based on the observed *F*_ST _values. Thus there is a low amount of gene flow between the two species (*N*_e_*m*, ranging from 0.7 to 0.9). These values are in line with other reported studies [5]. The estimates of gene flow among *An. arabiensis *populations indicate that there is a limited number of migrants between some populations (N_*e*_*m *ranging from 1.9 to 45; (Additional file 3), as would be expected for populations that are to some extent reproductively isolated.

**Figure 3:**
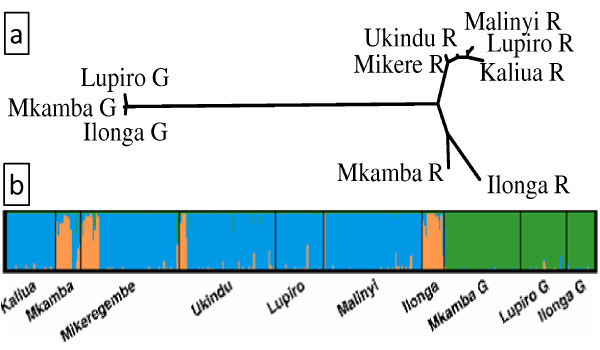
**Genetic distances and structuring**. (a) Unrooted neighbour-joining cladogram for *An. arabiensis *and *An. gambiae *populations based on *F*_ST _values. "GS" and "R" in each site indicate *An. gambiae s. s. *and *An. arabiensis *respectively. (b) Results from an individual level clustering analysis. The vertical bar indicates proportionate assignment of an individual to each cluster. Names of village sample sites are given below. "G" indicates *An. gambiae s. s*. The blue and green color denotes *An. arabiensis *and *An. gambiae s. s. *populations respectively and the pink color indicates an *An. arabiensis *subgroup.

The relatively large genetic level of differentiation between *An. arabiensis *populations within the valley can be visualized in the unrooted neighbour-joining tree, based on the matrix of pairwise *F*_ST _values (Figure 3a). Populations of *An. arabiensis *fall into two distinct clusters, with samples from all populations forming one cluster (cluster I) and populations from Mikeregembe, Mkamba and Ilonga forming the second cluster (Cluster II), (Figure 3b).

**Figure 4:**
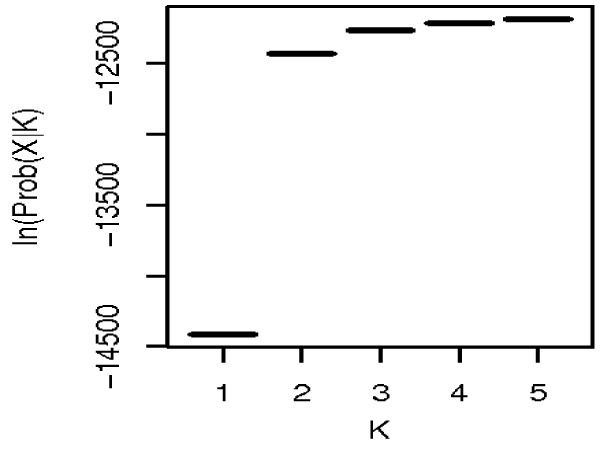
**Clustering analysis**. A bayesian clustering analysis using *structure *suggesting the possibility of structured population in *An. arabiensis*. The high numbers (probabilities) on the Y-axis corresponding to the pre-defined cluster (*K*) supports the possibility of a subpopulation at that cluster *(K*-value).

Bayesian clustering analysis was applied for all populations within and outside the Kilombero Valley. This approach is advantageous in that it does not require a prior population classification, but instead estimates the shared population ancestry based on observed genotypes, under the assumption of the presence of a H-W equilibrium and linkage equilibrium within each cluster [52]. Thus, based on the assumption that alleles occur in more than one population, individuals with unique alleles from all populations are placed into *K *clusters (chosen in advance), i.e. corresponding to the number of postulated Mendelian populations. As shown in Figure 4, solutions for hypothetical *An. arabiensis *population clusters from *K *= 4 and *K *= 5 showed a similar range of likelihood and the highest likelihood was observed at *K *= 3. Thus the most likely number of clusters (K) is three. This corresponds to three distinct genetic clusters: 1) The *An. gambiae s. s. *cluster 2) the *An. arabiensis *cluster I (present in all sites). and 3) the *An. arabiensis *cluster II (coexisting in Mkamba, Mikeregembe and Ilonga)

## Discussion

In this study, 13 microsatellite loci were used to describe the genetic structure of populations of *An. gambiae s.s. *and *An. arabiensis *in the Kilombero Valley, southern Tanzania. Both species occur in the Kilombero Valley with *An. arabiensis *more widespread. The *An. gambiae s.s. *populations sampled appear to represent a single genetic population, whereas *An. arabiensis *appear to occur as two discrete populations that are found in sympatry at some sites (for example, Mkamba, Ilonga and Mikeregembe, see Figure 3b). The higher genetic differentiation among *An. arabiensis *populations compared with that among *An. gambiae *populations is evidenced by (i) the genome-wide signature of departures from H-W equilibrium and high linkage disequilibrium between loci, (ii) higher *F*_ST _estimates between *An. arabiensis *than between *An. gambiae s.s. *populations, (iii) higher levels of gene flow between *An. gambiae s.s. *than *An. arabiensis *populations and (iv) Bayesian probability distribution at *K *= 3. This clearly indicates that *An. arabiensis *gene pool across the Kilombero Valley is not homogeneous. In contrast, *An. gambiae s. s. *showed low levels of genetic differentiation with high level of gene flow across populations, indicative of a homogeneous gene pool.

Population substructure observed in Ilonga, Mikeregembe and Mkamba, among *An. arabiensis *populations may be a consequence of several factors that need further investigations. Cytogenetic evidence suggests that *An. arabiensis *is an ancient species from which other members of the complex descended and that this species inhabited East Africa more than 6,000 yrs ago [14], indicative of a possibility for population structuring [8]. Consequently, the Kilombero Valley is surrounded by the ancient Eastern Arc mountain ranges (e.g., the Udzungwa and Mahenge mountains) providing stable wet climatic conditions [53] estimated to have existed for over 30 million yrs and this is where the two sampling sites (Ilonga and Mkamba, Figure 1) are located. Climatic stability as provided by the Eastern Arc mountains and the environmental heterogeneity maintained within it, may be among the major forces driving the genetic structure of *An. arabiensis *populations inhabiting Mkamba and Ilonga, as opposed to *An. arabiensis *populations outside and along the rest of the Kilombero Valley, such as Kaliua, Ukindu and Malinyi. It is important to note that Mikeregembe is a fishing camp [39] thus it is plausible that a subpopulation of *An. arabiensis *existing in Mkamba and Ilonga may have been introduced in Mikeregembe by fishermen from Mkamba (20 kms away) who regularly visit the camp for fishing activities. These mountains also create unequal climatic conditions across the Kilombero Valley which correlate very well with the abundance of the two malaria vector species [39,54]. For example, (i) *An. gambiae s.s *is commonly found in close proximity with forested Eastern Arc mountain areas, where the climate is stable and annual rainfall is higher than in the rest of the valley (> 2,000 mm/yr), (ii) *An. arabiensis *is commonly found in riverine areas where the climate is less stable and annual rainfall is lower (<1,200 mm/yr) [39]. Therefore, it is plausible that *An. arabiensis *populations inhabiting sites along the Eastern Arc mountains are subjected to a higher degree of environmental heterogeneity typical to these mountains, promoting genetic variation and the emergence of new adaptive genotypes [55].

Based on the difference in allele frequency (*F*_ST_), the level of genetic divergence between *An. gambiae s. s. *populations is lower (mean *F*_ST _= 0.006) than that between *An. arabiensis *populations (mean *F*_ST _= 0.066). The degree of divergence between *An. arabiensis *populations (mean *F*_ST _= 0.066) is even greater than the reported divergence between the well-known M and S forms of *An. gambiae s.s. *in West Africa (mean *F*_ST _= 0.035), [56]. From these observations it can be hypothesized that the *An. arabiensis *subpopulations are maintained by some degree of reproductive isolation. This hypothesis can also be supported by the absence of hybrids in sympatric *An. arabiensis *populations (e.g. in Mkamba, Mikeregembe and Ilonga; Figure 3b).

The *F*_ST _estimate reported in this study is higher than the reported *F*_ST _estimate for *An. arabiensis *from west, south and east Africa (0.035-0.038) where extensive *An. arabiensis *population differentiation was observed [25,27]. The *F*_ST _estimate observed in this study (0.066 - 0.100) is in line with the largest reported *An. arabiensis F*_ST _estimate (0.080-0.215). This is a comparison between mainland and island *An. arabiensis *populations [24], where gene flow is restricted by the ocean. Extensive *An. arabiensis *genetic differentiation was neither a result of any obvious physical barrier nor geographic distance, consistent with reports from other studies [26,27]. It can be deduced that factors other than physical barriers or geographical distance may be responsible for genetic differentiation in *An. arabiensis *[57]. In this study, highest genetic differentiation in *An. arabiensis *was recorded for markers located on chromosome X. 42% of tests involving markers on chromosome X deviated from Hardy-Weinberg equilibrium, in contrast to 0% in *An. gambiae s.s*. This does not only suggest the population to be structured, but also supports the hypothesis developed by Stump and colleagues that sex-linked differentiation is characterised by some degree of reproductive isolation mechanisms controlled by genes located in genomic regions within X chromosome [58].

The observed genetic *An. arabiensis *subgroups existing within the Kilombero Valley may be of great significance to malaria epidemiology. *Anopheles arabiensis *mosquitoes are reported to be phenotypicaly diverse. For example, *An. arabiensis *is more zoophilic and exophilic [59], and highly anthropophagic in other places [60]. Also *An. arabiensis *are able to tolerate higher temperatures and lower humidities than *An. gambiae s.s. *[61]. This phenomena, may prompt early biting activity for *An. arabiensis *and accelerate disease transmission. Therefore, if the observed population substructure is ignored, degrees of medically important phenotypes in *An. arabiensis *species, such as vectorial capacity, host preference and insecticide resistance, could be obfuscated. Thus, further investigations are needed to thoroughly understand the epidemiological significance of the observed population substructures.

## Conclusions

This study provides evidence that *An. arabiensis *in East Africa has a complex genetic structure with distinct populations occurring in sympatry, apparently maintained by some degree of reproductive isolation. It was hypothesized that ecological differences rather than a physical barrier or geographical distance are responsible for the observed *An. arabiensis *population substructure. Further investigation is needed not only to clearly establish the specific mechanisms underlying genetic differentiation in *An. arabiensis *populations, but also to identify the number of discrete malaria vector populations sustaining transmission along the Kilombero Valley, important for guiding the implementation of available and future control strategies. Proposed new genetic control strategies such as sterile insect technique (SIT) and genetically modified mosquitoes (GMM), require a homogeneous population structure for success. The observed level of genetic substructure may pose a challenge to the successful implementation of these approaches [62,63]. Since *An. arabiensis *populations appear to be maintained by some degree of reproductive isolation, multiple genetic modifications of *An. arabiensis *may be needed if genetic control is to be successful. Furthermore, the observed *An. arabiensis *population substructure may have different malaria transmission intensity, as populations with distinct genetic composition may have different vectorial capacities [64]. Further investigation of the association between the observed subpopulations and their susceptibility to malaria parasite infection, would be useful to explore if and how these genotypes could be influencing malaria transmission intensity along the Kilombero Valley.

The study showed that there may still be another level of genetic subdivision within the *An. gambiae *complex, suggesting the possibility of population expansion [65], with potential implication not only in understanding the evolutionary process of this complex but also for the application of vector control approaches. Further studies are recommended to investigate the spatial and temporal distribution of the observed genetic variation and population substructure in both *An. gambiae *and *An. arabiensis *along the Kilombero Valley.

## Competing interests

The authors declare that they have no competing interests.

## Authors' contributions

KRN, BGJK, GCL and HMF designed this study. KRN carried out the laboratory and SFS work. KRN and YL analysed and interpreted the data. KRN, HMF, GCL and BGJK drafted the manuscript. All authors read and approved the manuscript.

## Supplementary Material

Additional file 1 **P-values indicating statistical significance (*P *< 0.00044) of deviations from Hardy - Weinberg expectations for 13 microsatellite loci in populations of *An. arabiensis *and *An. gambiae s. s. *collected from within the Kilombero/Ulanga Valleys, Tanzania**. *Ag *= *An. gambiae s. s. *and *Aa *= *An. arabiensis*. Significant test at P ≤ 0.00044 are in bold. Click here for file

Additional file 2 **Pairwise estimates of genetic divergence (*F***_**ST**_**) between An. *gambiae *and *An. arabiensis *in the Kilombero Valley**. The total numbers of mosquitoes screened in each location are in brackets. Underlined values indicate inter-species comparison between *An. gambiae *and *An. arabiensis*. The non-underlined values indicate intra-species comparisons, i.e. within *An. gambiae *and *An. arabiensis*. Values in italics are statistically significant. Significance levels at ^¥^P < 0.05, ^$^P < 0.01 and *P < 0.001. '*Ag*' = *An. gambiae s. s. *and '*Aa' *= *An. arabiensis*. Click here for file

Additional file 3 **Estimated number of migrants between *An. gambiae s. l. *populations within and outside Kilombero Valley (Kaliua)**. 'Ag' stands for *An. gambiae s. s. *and 'Aa' stands for *An. arabiensis*. Click here for file
