# Supplementary material for: Population genetic structure of Anopheles arabiensis and Anopheles gambiae in a malaria endemic region of southern Tanzania
Source: Malar J. 2011 Oct 5;10:289. doi: 10.1186/1475-2875-10-289 (PMC3195206; doi:10.1186/1475-2875-10-289)
Supplement: Additional file 1 — P-values indicating statistical significance (P < 0.00044) of deviations from Hardy - Weinberg expectations for 13 microsatellite loci in populations of An. arabiensis and An. gambiae s. s. collected from within the Kilombero/Ulanga Valleys, Tanzania. Ag = An. gambiae s. s. and Aa = An. arabiensis. Significant test at P ≤ 0.00044 are in bold. [file 1475-2875-10-289-S1.PDF]

Table 1: P-values indicating statistical significance (*P* < 0.00044) of deviations from Hardy – Weinberg expectations for 13 microsatellite loci in populations of *An. arabiensis* and *An. gambiae s. s.* collected from within the Kilombero/Ulanga Valleys, Tanzania. N/A = fixed alleles, *Ag* = *An. gambiae s. s.* and *Aa* = *An. arabiensis*. Significant test at P ≤ 0.00044 are in bold.

	An. arabiensis							*An gambiae s. s.*		
Chromosome/Locus	Ilonga (Aa)	Malinyi (Aa)	Lupiro (Aa)	Ukindu (Aa)	Mikeregembe (Aa)	Mkamba (Aa)	Kaliua (Aa)	Ilonga (Ag)	Lupiro (Ag)	Mkamba (Ag)
X AGXH100	0.02719	**0.00000**	0.01362	**0.00000**	**0.00000**	1.00000	0.00198	0.52185	0.02420	0.12993
AGXH25	0.03480	**0.00000**	**0.00001**	**0.00000**	**0.00000**	**0.00014**	**0.00000**	0.05385	0.05672	0.78633
AGXH71	0.00171	0.01108	0.25532	0.01049	0.00982	0.00270	0.01200	0.00057	0.23734	0.16610
2 AG2H85	0.05530	0.14529	0.37553	**0.00006**	**0.00014**	1.00000	**0.00029**	0.94075	0.00507	0.28175
AG2H164	**0.00006**	0.07883	**0.00000**	0.00586	0.01234	0.00857	0.28081	**0.00009**	**0.00000**	**0.00000**
AG2H175	0.79302	0.22363	0.54858	0.30838	0.42998	0.36502	1.00000	**0.00000**	**0.00000**	**0.00000**
AG2H197	0.08331	0.02250	0.31518	**0.00006**	**0.00000**	0.25521	0.83194	0.00430	0.48666	0.51077
AG2H675	1.00000	0.48918	0.01798	0.17940	0.46848	0.52015	0.54126	0.50763	0.30074	0.02176
3 AG3H127	N/A	N/A	N/A	N/A	N/A	N/A	N/A	0.00078	0.27888	**0.00003**
AG3H249	0.16835	0.07418	0.06044	0.22735	0.00259	0.31076	0.23614	0.67873	0.05164	0.19956
AG3H311	N/A	N/A	1.00000	N/A	N/A	N/A	N/A	**0.00000**	**0.00000**	**0.00000**
AG3H811	**0.00004**	**0.00000**	0.02007	**0.00000**	**0.00021**	**0.00010**	0.51587	0.28928	0.47032	0.01804
AG3H93	1.00000	0.30044	0.42682	**0.00000**	0.19691	0.02498	0.00926	0.04169	0.97865	0.13654
