# Supplementary material for: Population genetic structure of Anopheles arabiensis and Anopheles gambiae in a malaria endemic region of southern Tanzania
Source: Malar J. 2011 Oct 5;10:289. doi: 10.1186/1475-2875-10-289 (PMC3195206; doi:10.1186/1475-2875-10-289)
Supplement: Additional file 2 — Pairwise estimates of genetic divergence (FST) between An. gambiae and An. arabiensis in the Kilombero Valley. The total numbers of mosquitoes screened in each location are in brackets. Underlined values indicate inter-species comparison between An. gambiae and An. arabiensis. The non-underlined values indicate intra-species comparisons, i.e. within An. gambiae and An. arabiensis. Values in italics are statistically significant. Significance levels at ¥P < 0.05, $P < 0.01 and *P < 0.001. 'Ag' = An. gambiae s. s. and 'Aa' = An. arabiensis. [file 1475-2875-10-289-S2.PDF]

Table 2: Pairwise estimates of genetic divergence (F_{ST}) between An. *gambiae* and *An. arabiensis* in the Kilombero Valley. The total numbers of mosquitoes screened in each location are in brackets. Underlined values indicate inter-species comparison between *An. gambiae* and *An. arabiensis.* The non-underlined values indicate intra-species comparisons, i.e. within *An. gambiae* and *An. arabiensis*. Values in italics are statistically significant. Significance levels at $^{¥}$P < 0.05, $^{\$}$P< 0.01 and *P<0.001. '*Ag*' = *An. gambiae s. s.* and '*Aa*' = *An. arabiensis*

	Ilonga (Aa)	Malinyi (Aa)	Lupiro (Aa)	Ukindu (Aa)	Mikeregembe (Aa)	Mkamba (Aa)	Kaliua (Aa)	Ilonga (Ag)	Lupiro (Ag)
Ilonga (15)	-	-	-	-	-	-	-	-	-
Malinyi (64)	_0.1031*_	-	-	-	-	-	-	-	-
Lupiro (32)	_0.0998*_	_0.0055_	-	-	-	-	-	-	-
Ukindu (64)	_0.0837*_	_0.0084_ $^{¥}$	_0.0157_ $^{\$}$	-	-	-	-	-	-
Mikeregembe (64)	_0.0731*_	_0.0205*_	_0.0241*_	_0.0129_ $^{\$}$	-	-	-	-	-
Mkamba (17)	0.0143	_0.0391*_	_0.0374*_	_0.0250_ $^{\$}$	_0.0273_ $^{\$}$	-	-	-	-
Kaliua (32)	_0.1191*_	_0.0125_ $^{¥}$	_0.0181_ $^{\$}$	_0.0127_ $^{¥}$	_0.0265*_	_0.0479*_	-	-	-
Ilonga-Ag (18)	_0.2471*_	_0.2611*_	_0.2393*_	_0.2378*_	_0.2453*_	_0.2193*_	_0.2645*_	-	-
Lupiro-Ag (30)	_0.2508*_	_0.2625*_	_0.2410*_	_0.2384*_	_0.2443*_	_0.2273*_	_0.2661*_	0.0109	-
Mkamba-Ag (50)	_0.2393*_	_0.2578*_	_0.2340*_	_0.2333*_	_0.2399*_	_0.2164*_	_0.2569*_	0.0029	0.0071 $^{¥}$
