# Supplementary material for: Population genetic structure of Anopheles arabiensis and Anopheles gambiae in a malaria endemic region of southern Tanzania
Source: Malar J. 2011 Oct 5;10:289. doi: 10.1186/1475-2875-10-289 (PMC3195206; doi:10.1186/1475-2875-10-289)
Supplement: Additional file 3 — Estimated number of migrants between An. gambiae s. l. populations within and outside Kilombero Valley (Kaliua). 'Ag' stands for An. gambiae s. s. and 'Aa' stands for An. arabiensis. [file 1475-2875-10-289-S3.PDF]

Table 3: Estimated number of migrants between *An. gambiae s. l.* populations within and outside Kilombero Valley (Kaliua). 'Ag' stands for *An. gambiae s. s.* and 'Aa' stands for *An. arabiensis.*

Villages	Ilonga (Aa)	Malinyi (Aa)	Lupiro (Aa)	Ukindu (Aa)	Mikeregembe (Aa)	Mkamba (Aa)	Kaliua (Aa)	Ilonga (Ag)	Lupiro (Ag)
Ilonga (Ag)	0.8	0.7	0.8	0.8	0.7	0.9	0.7	-	-
Lupiro (Aa)	2.3	45	-	-	-	-	-	-	-
Lupiro (Ag)	0.8	0.7	0.8	0.8	0.7	0.9	0.7	22.5	-
Mkamba (Aa)	17.2	6.2	6.4	9.4	8.9	-	-	-	-
Mkamba (Ag)	0.8	0.7	0.8	0.8	0.7	0.9	0.7	85.9	34.6
Kaliua (Aa)	1.9	19.8	13.6	19.4	9.2	5	-	-	-
